# Synergistic white matter protection with acute-on-chronic endotoxin and subsequent asphyxia in preterm fetal sheep

**DOI:** 10.1186/1742-2094-11-89

**Published:** 2014-05-16

**Authors:** Lotte G van den Heuij, Sam Mathai, Joanne O Davidson, Christopher A Lear, Lindsea C Booth, Mhoyra Fraser, Alistair J Gunn, Laura Bennet

**Affiliations:** 1 Department of Physiology, University of Auckland, Park Road, Grafton, Auckland 1023, New Zealand; 2 The Liggins Institute, University of Auckland, Park Road, Grafton, Auckland 1023, New Zealand

**Keywords:** Asphyxia, Fetus, Inflammation, Lipopolysaccharide, Postconditioning, Preconditioning

## Abstract

**Background:**

Perinatal asphyxia and exposure to intrauterine infection are associated with impaired neurodevelopment in preterm infants. Acute exposure to non-injurious infection and/or inflammation can either protect or sensitize the brain to subsequent hypoxia-ischemia. However, the effects of subacute infection and/or inflammation are unclear. In this study we tested the hypothesis that acute-on-chronic exposure to lipopolysaccharide (LPS) would exacerbate white matter injury after subsequent asphyxia in preterm fetal sheep.

**Methods:**

Fetal sheep at 0.7 gestational age received a continuous LPS infusion at 100 ng/kg for 24 hours, then 250 ng/kg/24 hours for 96 hours, plus 1 μg boluses of LPS at 48, 72, and 96 hours or the same volume of saline. Four hours after the last bolus, complete umbilical cord occlusion or sham occlusion was induced for 15 minutes. Sheep were sacrificed 10 days after the start of infusions.

**Results:**

LPS exposure was associated with induction of microglia and astrocytes and loss of total and immature and mature oligodendrocytes (n = 9) compared to sham controls (n = 9). Umbilical cord occlusion with saline infusions was associated with induction of microglia, astrogliosis, and loss of immature and mature oligodendrocytes (n = 9). LPS exposure before asphyxia (n = 8) was associated with significantly reduced microglial activation and astrogliosis and improved numbers of immature and mature oligodendrocytes compared to either LPS exposure or asphyxia alone.

**Conclusions:**

Contrary to our initial hypothesis, the combination of acute-on-chronic LPS with subsequent asphyxia reduced neuroinflammation and white matter injury compared with either intervention alone.

## Introduction

Preterm birth occurs in 5 to 10% of all live births and is associated with considerable risk of longterm neurodevelopmental disability [1]. The precise causes of longterm maldevelopment are controversial, but there is increasing evidence that diffuse injury in the white matter tracts is associated with astrocytosis and maturational arrest of oligodendrocytes at postmortem, and longterm neurobehavioral disturbances and intellectual disabilities [2,3]. Exposure to perinatal hypoxia-ischemia (HI) or infection and/or inflammation are both associated with adverse neural outcomes in preterm infants [4-7], and there is highly suggestive evidence that the combination of infection and/or inflammation and HI may be particularly deleterious [8].

There is strong evidence in neonatal rodents that an injection of a non-damaging dose of gram negative lipopolysaccharide (LPS) given either within 6 hours or more than 72 hours before HI can dramatically increase white and gray matter injury [9-13]. In contrast, an injection of LPS at intermediate times, such as 24 hours before HI, reduced subsequent neural injury (preconditioned the brain) [12,14,15]. There is evidence that dose as well as timing is important, since whereas low-dose LPS reduced damage from subsequent HI 24 hours later, higher dose LPS (0.3 mg/kg) was associated with increased inflammation and mortality [14]. There are few data available in large animals. In preterm fetal sheep, high-dose LPS (50 μg/kg) given one hour before occlusion of the maternal aorta for two minutes impaired circulatory centralization and cerebral oxygen delivery, with increased mortality [16]. It is striking though, that these studies have all involved single boluses of LPS. In modern neonatal care, subclinical infection is more common than acute severe sepsis and yet is also associated with adverse outcomes [5,6].

We have recently reported that in preterm fetal sheep 48 hours exposure to a low-dose chronic infusion of LPS, that did not cause any hemodynamic disturbance, before three boluses of high-dose LPS 24 hours apart significantly reduced mortality and attenuated the nadir of hypotension after the LPS boluses [17,18]. This paradigm of acute-on-chronic LPS was associated with a fetal inflammatory response and white matter injury. The effect of such ongoing exposure to infection on the susceptibility of the immature brain to subsequent HI is unknown.

In this study, we tested the hypothesis that acute-on-chronic inflammation would sensitize the developing brain to a subsequent period of asphyxia [19], induced by 15 minutes of umbilical cord occlusion in preterm fetal sheep at 0.7 gestational age. This duration of asphyxia is associated with moderate neural injury [19] and was chosen to allow scope to show modulation of injury by LPS. In view of the evidence discussed above that a four-hour delay after acute LPS exposure typically increases HI injury in neonatal animals [12], asphyxia was induced four hours after the last bolus of high-dose LPS. We have previously shown that a similar paradigm of acute-on-chronic LPS exposure was associated with more rapid chemoreflex adaptation to umbilical cord occlusion and did not compromise fetal hemodynamic adaptation [18]. The neural maturation of 0.7 ga fetal sheep is broadly equivalent to between 28 and 32 weeks of human development [20].

## Methods

### Animal surgery

We have previously reported the cardiovascular, hemodynamic, and white matter changes in an overlapping subset of sham controls and LPS-treated animals [17]. All procedures were approved by the Animal Ethics Committee of the University of Auckland. In brief, 35 time-mated Romney–Suffolk-cross fetal sheep were instrumented using sterile technique at 97 to 98 days ga (full term is 145 days). Food but not water was withdrawn 18 hours before surgery. Ewes were given 1 mL/10 kg oxytetracycline (200 mg/mL, Phoenix Pharm Distributors Ltd., Auckland, New Zealand) intramuscularly for prophylaxis 30 minutes before the start of surgery. Ewes were anesthetized by an intravenous injection of propofol (5 mg/kg, AstraZeneca Limited, Auckland, New Zealand), followed by between 2 and 3% isoflurane in oxygen. The depth of anesthesia and maternal respiration were constantly monitored by trained anesthetic staff. Ewes received a constant infusion of an isotonic saline drip (at an infusion rate of approximately 250 mL/h) to maintain fluid balance.

Following a maternal midline abdominal incision and exteriorization of the fetus from the uterus, a femoral and brachial artery and a brachial vein were catheterized with polyvinyl catheters to allow for mean arterial blood pressure monitoring and preductal blood sampling and infusions. An amniotic catheter was secured to the fetal shoulder. Electrocardiogram (ECG) electrodes (Cooner Wire Co., Chatsworth, California, United States) were sewn across the fetal chest to record fetal heart rate. An inflatable silicon occluder was placed around the umbilical cord (In Vivo Metric, Healdsburg, California, United States). The uterus was then closed. Antibiotics (80 mg gentamicin, Pharmacia and Upjohn, Rydalmere, New South Wales, Australia) were administered into the amniotic sac. The maternal laparotomy skin incision was infiltrated with 10 mL of a local analgesic containing 0.5% bupivacaine plus adrenaline (AstraZeneca Ltd., Auckland, New Zealand). All fetal catheters and leads were exteriorized through the maternal flank. The maternal long saphenous vein was catheterized for postoperative maternal care and euthanasia.

### Postoperative care

Sheep were housed together in separate metabolic cages with access to food and water *ad libitum*. They were kept in a temperature-controlled room (16 ± 1°C, humidity 50 ± 10%), in a 12 hour light/dark cycle. Antibiotics were intravenously given daily for four days to the ewe (600 mg benzylpenicillin, Novartis Ltd, Auckland, New Zealand, and 80 mg gentamicin, Pharmacia and Upjohn). Fetal catheters were continuously infused with heparinized saline (20 U/mL at 0.15 mL/hour) and the maternal catheter was maintained by daily flushing.

### Experimental design

Experiments started five days after surgery at 102 ± 0 days of gestational age. Fetuses were randomized into four experimental groups: (1) Chronic saline infusion and saline boluses plus sham asphyxia (Sal-Sham; n = 9). (2) Chronic LPS infusion plus three LPS boluses plus sham asphyxia (LPS-Sham; n = 9). (3) Chronic saline infusion and saline boluses plus asphyxia for 15 minutes (Sal-Asp; n = 9). (4) Chronic LPS infusion plus three LPS boluses plus asphyxia (LPS-Asp; n = 8). LPS was dissolved in saline and infused at a rate of 100 ng/24 hours for the first 24 hours, then increased to 250 ng/24 hours for the next 96 hours. LPS and saline boluses (1 μg/mL) were given at 48, 72, and 96 hours after the start of infusions.

Asphyxia was induced by rapid inflation of the silicon occluder for 15 minutes with a volume of saline known to completely occlude the umbilical cord, four hours after the third saline or LPS bolus. Successful occlusion was confirmed by the rapid onset of bradycardia and by subsequent changes in pH and blood gases [21,22].

Fetal arterial blood samples were collected daily, starting from 24 hours before the start of the saline or LPS infusions, plus 6 hours after each bolus of LPS or saline. During umbilical cord occlusion, fetal arterial blood was additionally taken at 5 and 12 minutes during occlusion and 10 minutes and two hours after occlusion. Blood samples were analyzed for pH, blood gases (845 Blood Gas Analyzer/Co-oximeter, Ciba-corning Diagnostics, Massachusetts, United States), and glucose and lactate measurements (YSI 2300, Yellow Springs Instruments, Yellow Springs, Ohio, United States). Additional plasma was collected for cytokine and cortisol measurements.

### Fetal cytokine measurements

Cytokine levels in the plasma were measured using in-house enzyme-linked immunosorbent assays [17]. Interleukin (IL)-6 was detected using antibodies specific to ovine IL-6 (Epitope Technologies, Melbourne, Australia). Standards were ovine recombinant IL-6 (Protein Express, Cincinnati, Ohio, United States). The standard series ranged from between 0 and 5 ng/mL. The assay sensitivity was 0.097 ng/mL and internal quality controls were included in each assay. Cytokine concentrations were within the detection limit in all samples. IL-10 was detected using antibodies specific to the bovine species (AbD Serotec, Oxford, United Kingdom) [17]. Standards used were recombinant bovine IL-10 (kindly supplied by Professor G Entrican, Moredun Research Institute, Scotland) and ranged between 0 and 11 BU/mL with a detection sensitivity of 0.086 BU/mL.

### Fetal cortisol

Fetal plasma cortisol levels were measured using triple quadrupole mass spectrometry [17]. A total of 100 μL of internal standard (20 ng/mL cortisol-d4 in water) was added to 200 μL plasma. Steroids were extracted using 1 mL of ethyl acetate (Merck KGaA, Darnstadt, Germany). After removal of the organic supernatant, samples were dried by vacuum concentration (Savant SC250EXP, Thermo Scientific, Asheville, North Carolina, United States), re-suspended in 60 μL of mobile phase 72% methanol (Merck KGaA) and 28% water, and transferred to HPLC injector vials. A total of 12 μL was injected onto an HPLC mass spectrometer system consisting of an Accela MS pump and autosampler followed by an Ion Max APCI source on a Finnigan TSQ Quantum Ultra AM triple quadrupole mass spectrometer, all controlled by Finnigan Xcaliber software (Thermo Electron Corporation, San Jose, California, United States). The mobile phase was isocratic, flowing at 250 μL/min through a Luna HST 2.6 μm C18 (2) 100 × 3.0 mm column at 40°C (Phenomenex, Auckland, New Zealand). Retention time was 3.1 minutes for both cortisol and cortisol-d4. Ionization was in positive mode and Q2 had 1.2 mTorr of argon. The mass transitions followed were: cortisol-d4 367.2 – 121.2 at 28 V and cortisol 363.2 – 122.2 at 28 V. Mean inter- and intra-assay coefficient of variation values for cortisol were 5.8 and 6.0% respectively.

### Postmortem and tissue preparation

**Figure 1:**
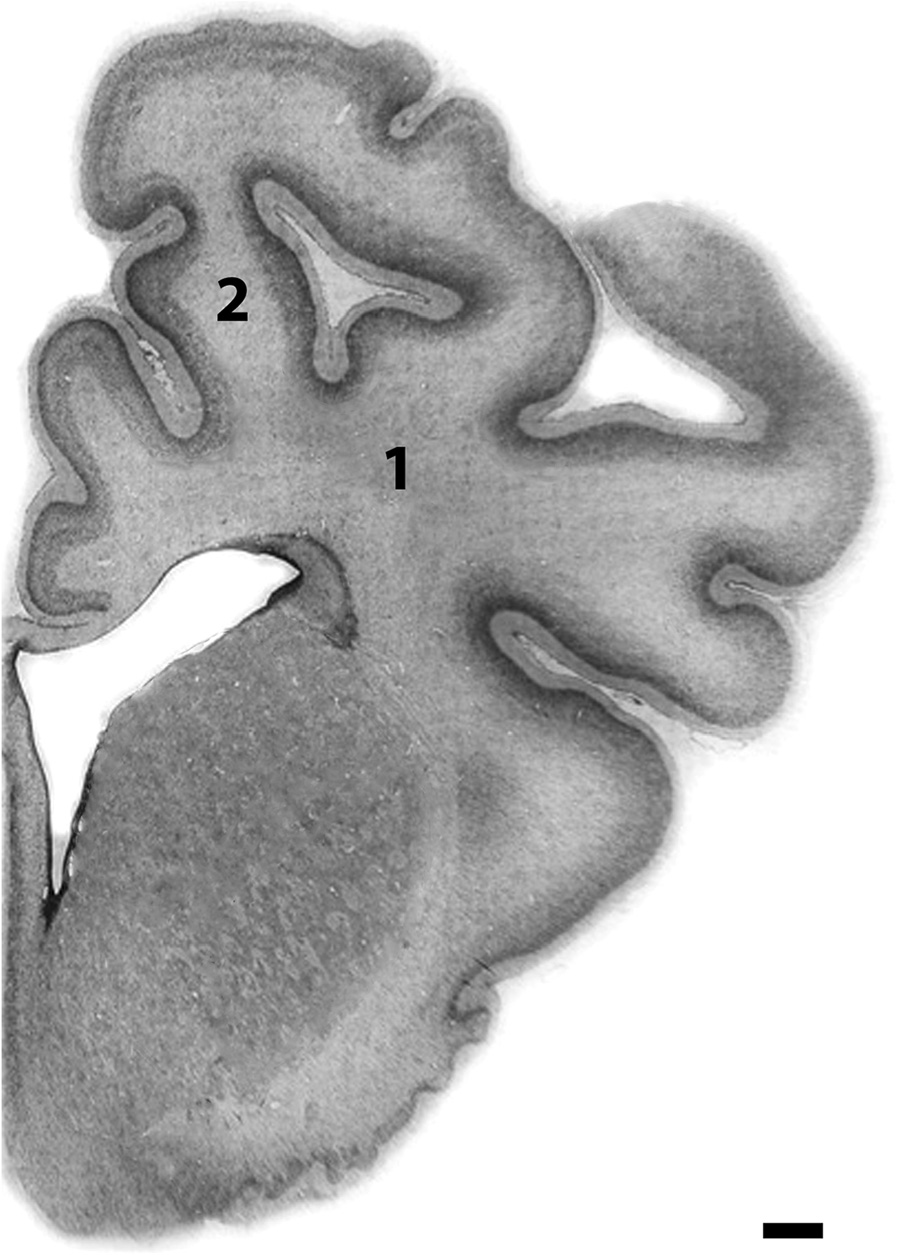
**Photomicrographs of coronal sections of fetal sheep brain showing the white matter regions analyzed in this study.** 1) periventricular white matter, 2) intragyral white matter. Scale bar = 2.5 mm.

Ewes were euthanized after day 10 with maternal intravenous sodium pentobarbitone (9 g, Pentobarb 300, Chemstock International, Christchurch, New Zealand). Fetuses were quickly removed from the uterus and their brains were perfusion fixed *in situ* with 500 mL saline, followed by 4% neutral buffered formalin infused by gravity feed, and post fixed in formalin for one week before processing and paraffin embedding. Coronal sections measuring 10 μm were cut at the level of mid striatum (approximately 17 mm anterior to stereotaxic zero, shown in Figure 1) and hippocampus (approximately 26 mm anterior to stereotaxic zero) [23] for immunohistochemistry.

### Immunohistochemistry

Deparaffinized and rehydrated sections were antigen-retrieved in a citrate buffer (pH 6.0) for 20 minutes using a pressure cooker method (2100 Retriever, Prestige Medical Ltd; Blackburn, United Kingdom). Phosphate-buffered saline (PBS) washed sections were then treated with 1% hydrogen peroxide in methanol for 30 minutes in the dark for quenching endogenous peroxidase activity. Blocking was done with 3% goat/horse serum in PBS for one hour at room temperature. Primary and secondary antibodies were diluted in 3% goat/horse serum in PBS. Sections were incubated with primary antibodies at 4°C overnight for immunohistochemical labeling. Reactive microglia were labelled with 1:200 goat anti-ionized calcium-binding adapter molecule-1 antibody (Iba-1, Sapphire Bioscience Ltd., Auckland, New Zealand). Cells expressing inflammatory cytokines were labelled with 1:200 monoclonal mouse anti-tumor necrosis factor-α (TNF-α) (Abacus ALS, Auckland, New Zealand). Reactive astrocytes were labelled with 1:500 mouse anti-glial fibrillary acidic protein (GFAP) (Chemicon International Inc., Temecula, California, United States). Cells undergoing apoptosis were labelled with 1:200 rabbit anti-caspase-3 ASP175 (Cell Signaling Technology, Danvers, Massachusetts, United States). Immature and mature oligodendrocytes were labelled with 1:200 mouse monoclonal anti-2′, 3′-cyclic nucleotide 3′-phosphodiesterase (CNPase) (Chemicon International Inc.). Rabbit anti-oligodendrocyte transcription factor-2 (Olig2) (Merck-Millipore, Manukau City, New Zealand) in a dilution of 1:200 was used as a marker of all cells in the oligodendrocyte lineage [24]. After overnight incubation with corresponding secondary antibodies (1:200) at 4°C, followed by 1:200 ExtrAvidin (Sigma-Aldrich Ltd., Auckland, New Zealand) at room temperature for three hours, sections were treated with SIGMAFAST™ 3,3′ diaminobenzidine (Sigma-Aldrich Ltd.) to visualize immunoreactivity and mounted with di-n-butyl phthalate in xylene (Sigma-Aldrich Ltd.). Negative controls were run in parallel.

### Assessment of brain injury

Inflammatory responses, CNPase, and Olig2-positive oligodendrocytes and apoptotic cells were quantified in the periventricular white matter (PVWM) and intragyral white matter (IGWM) of the first parasagittal gyrus by investigators blinded to the study groups. The area of microglia infiltration and cell counts were measured by light microscopy with a Nikon 80i light microscope (Scitech Ltd., Preston, Victoria, Australia), using StereoInvestigator software (v10, Microbrightfield Bioscience (MBF), Williston, Vermont, United States).

Sampling was undertaken using stereological principles by tracing the contour of the region of interest and then randomly translating a grid on the image and applying a fractionator probe with a counting frame for object exclusion or inclusion at 40 × magnification. A total of 30 sites were screened per slide (counting frame size 100 × 100 μm) and all the estimated cell counts were converted to cell density (cells/mm^2^) by the equation: estimated total counts by fractionator divided by contour area (μm^2^) × 10^6^. In the case of cells undergoing apoptosis, manual counting was done using the entire region. Values for the left and right hemispheres and for the two histological levels per animal were averaged.

### Data analysis and statistics

Data were compared between groups using ANOVA followed by the least significant difference test *post hoc* tests when a significant effect of group was found (SPSS v22, SPSS Inc., Chicago, Illinois, United States). For analysis of immunohistochemical findings, region was treated as a repeated measure. Statistical significance was accepted at *P* <0.05. Data are mean ± standard error of the mean (SEM).

## Results

There was no mortality in any of the experimental groups. The LPS-Asp group had lower body weights than the other groups: Sal-Sham 2.10 ± 0.09 kg, LPS-Sham 2.27 ± 0.09 kg, and Sal-Asp 2.22 ± 0.10 kg versus LPS-Asp 1.82 ± 0.08 kg (*P* <0.01). There was no significant difference in brain weight between any of the groups: Sal-Sham 29.7 ± 2.3 g*.* LPS-Sham 27.2 ± 1.1 g, Sal-Asp 30.4 ± 1.4 g, and LPS-Asp 28.2 ± 2.0 g.

### Fetal blood composition

There were no significant differences in baseline pH, blood gas, glucose, or lactate measurements between groups (Table 1). Chronic LPS infusion was not associated with any significant differences between groups, except for an increase in glucose in the Sal-Sham group on day two (*P* <0.05). The first LPS bolus was associated with a significant reduction in glucose in both LPS-exposed groups compared to both the Sal-Sham and Sal-Asp groups (*P* <0.05). The second LPS bolus was associated with a significant increase in lactate in the LPS-Sham group compared to the Sal-Sham group following the third bolus and period of asphyxia (*P* <0.05). There was a significant increase in lactate in both asphyxia groups and a significant increase in glucose in the Sal-Asp group compared to Sal-Sham (Table 2, *P* <0.05). All changes had resolved by day six.

**Table 1:** Arterial pH, blood gases, glucose, and lactate levels

	**Group**	**-24 h**	**Day 1**	**Day 1 + 6 h**	**Day 2**	**Day 3**	**Day 3 + 6 h**	**Day 4 + 6 h**	**Day 5 + 6 h**	**Day 6**
**pH**	S-S	7.38 ± 0.00	7.37 ± 0.01	7.37 ± 0.00	7.36 ± 0.00	7.36 ± 0.01	7.36 ± 0.00	7.35 ± 0.01	7.35 ± 0.01	7.35 ± 0.01
	L-S	7.38 ± 0.00	7.38 ± 0.00	7.38 ± 0.00	7.38 ± 0.00	7.38 ± 0.00	7.34 ± 0.00	7.36 ± 0.00	7.36 ± 0.00	7.35 ± 0.00
	S-A	7.36 ± 0.00	7.37 ± 0.00	7.36 ± 0.00	7.37 ± 0.00	7.36 ± 0.00	7.37 ± 0.00	7.36 ± 0.00	7.36 ± 0.01	7.36 ± 0.00
	L-A	7.37 ± 0.00	7.37 ± 0.00	7.37 ± 0.00	7.36 ± 0.00	7.36 ± 0.00	7.37 ± 0.00	7.38 ± 0.00*#	7.37 ± 0.00	7.35 ± 0.01
**paCO**_**2**_ (mmHg)	S-S	45.4 ± 1.4	49.5 ± 0.8	49.6 ± 1.1	50.1 ± 1.0	50.1 ± 1.5	48.3 ± 1.1	49.3 ± 1.2	50.0 ± 2.0	48.3 ± 1.4
	L-S	43.9 ± 1.6	49.2 ± 1.6	48.7 ± 1.0	49.2 ± 1.5	48.9 ± 1.1	54.8 ± 2.1	50.7 ± 1.6	48.5 ± 1.4	49.1 ± 1.1
	S-A	43.5 ± 1.5	46.5 ± 1.2	47.7 ± 1.0	50.0 ± 1.3	48.5 ± 0.9	49.2 ± 0.8	48.3 ± 1.0	46.0 ± 1.0	47.8 ± 1.7
	L-A	42.6 ± 1.1	46.6 ± 1.3	46.2 ± 1.6	48.0 ± 1.7	48.2 ± 1.5	49.3 ± 1.8	46.5 ± 1.2#	43.9 ± 1.6*#	47.4 ± 1.5
**paO**_**2**_ (mmHg)	S-S	24.6 ± 1.2	25.2 ± 1.5	24.0 ± 1.6	24.6 ± 2.3	26.3 ± 2.4	23.2 ± 2.9	24.3 ± 3.2	24.7 ± 2.6	25.3 ± 2.3
	L-S	26.4 ± 1.2	26.0 ± 1.7	25.4 ± 1.4	25.9 ± 1.7	25.0 ± 2.0	21.4 ± 1.6	24.2 ± 2.7	25.9 ± 2.5	24.7 ± 1.9
	S-A	25.6 ± 0.8	24.9 ± 1.5	24.7 ± 1.3	24.3 ± 1.5	24.1 ± 1.7	24.3 ± 1.8	24.3 ± 1.4	26.2 ± 1.3	25.3 ± 1.1
	L-A	27.2 ± 0.8	25.4 ± 0.7	24.6 ± 1.0	26.2 ± 0.9	25.3 ± 0.8	22.6 ± 0.8	21.3 ± 1.3	24.2 ± 1.30	27.6 ± 1.6
**Lactate** (mmol/L)	S-S	0.8 ± 0.1	0.8 ± 0.1	0.8 ± 0.1	0.8 ± 0.0	0.8 ± 0.06	0.81 ± 0.1	0.8 ± 0.1	0.9 ± 0.1	0.8 ± 0.1
	L-S	0.7 ± 0.1	0.7 ± 0.1	0.7 ± 0.0	0.7 ± 0.0	0.8 ± 0.08	1.50 ± 0.2	1.5 ± 0.4*	0.7 ± 0.0	0.6 ± 0.1
	S-A	0.8 ± 0.1	0.8 ± 0.1	0.9 ± 0.1	0.9 ± 0.1	0.9 ± 0.13	1.1 ± 0.16	0.9 ± 0.1#	2.2 ± 0.4*#	0.8 ± 0.1
	L-A	0.6 ± 0.1	0.6 ± 0.1	0.7 ± 0.1	0.7 ± 0.1	0.7 ± 0.05	1.1 ± 0.31	0.8 ± 0.1	1.8 ± 0.4#	0.7 ± 0.1
**Glucose** (mmol/L)	S-S	0.8 ± 0.1	1.1 ± 0.1	1.2 ± 0.1	1.3 ± 0.1	1.2 ± 0.1	1.3 ± 0.1	1.2 ± 0.1	1.2 ± 0.1	1.2 ± 0.1
	L-S	0.8 ± 0.1	0.8 ± 0.1	0.9 ± 0.1	1.0 ± 0.1*	1.1 ± 0.1	0.9 ± 0.1*	1.0 ± 0.1	1.0 ± 0.1	0.9 ± 0.1
	S-A	1.0 ± 0.1	0.9 ± 0.1	1.2 ± 0.1	1.1 ± 0.1*	1.1 ± 0.0	1.3 ± 0.1#	1.2 ± 0.1	1.5 ± 0.1*	1.0 ± 0.1
	L-A	0.8 ± 0.1	1.0 ± 0.2	0.9 ± 0.1	1.1 ± 0.1*	1.1 ± 0.1	0.8 ± 0.1*§	1.0 ± 0.1	1.2 ± 0.1	1.1 ± 0.1

S-S = Sal-Sham (saline infusion + saline boluses + sham occlusion); L-S = LPS-Sham (LPS infusion + LPS boluses + sham occlusion); S-A = Sal-Asp (saline infusion + saline boluses + 15 minutes of umbilical cord occlusion); L-A = LPS-Asp (LPS infusion + LPS boluses + 15 minutes of umbilical cord occlusion). Saline or LPS infusions were started on day 1; boluses of saline or LPS were given on days 3, 4 and 5. Data during and after umbilical cord occlusion performed on day 5, 4 hours after the last LPS or saline bolus, are shown in Table 2. The chronic low-dose LPS or saline infusion continued from time zero to the end of day 5. Days 7 to 10 are not shown as there were no differences between groups at those times. LPS, lipopolysaccharide; paCO_2_, fetal arterial pressure of CO_2_ (mmHg); paO_2_, fetal arterial pressure of oxygen (mmHg). * *P* <0.05 versus Sal-Sham, # *P* <0.05 versus LPS-Sham, § *P* <0.05 versus Sal-Asp. Data are mean ± SEM.

**Table 2:** Arterial pH, blood gases, lactate, and glucose values before during and after umbilical cord occlusion

	**Group**	**Baseline**	**5 min**	**12 min**	**10 min post**	**2 h post**
**pH**	S-A	7.35 ± 0.00	7.02 ± 0.01	6.92 ± 0.00	7.21 ± 0.00	7.36 ± 0.00
	L-A	7.36 ± 0.00	6.98 ± 0.01§	6.90 ± 0.00	7.22 ± 0.00	7.36 ± 0.00
**paCO**_**2**_ (mmHg)	S-A	48 ± 1.1	90.2 ± 2.9	111.4 ± 3.1	45.3 ± 0.3	46.5 ± 0.8
	L-A	47.6 ± 1.2	95.7 ± 4.7	108.6 ± 6.5	46.4 ± 1.4	49.2 ± 0.9
**paO**_**2**_ (mmHg)	S-A	24.6 ± 0.8	8.5 ± 0.9	8.1 ± 1.1	34.6 ± 1.3	25.1 ± 0.8
	L-A	24.8 ± 0.8	7.1 ± 0.4	9.4 ± 0.6	33.1 ± 1.0	25 ± 0.9
**Lactate** (mmol/L)	S-A	0.87 ± 0.1	3.4 ± 0.2	4.6 ± 0.3	4.3 ± 0.5	1.2 ± 0.3
	L-A	0.55 ± 0.1	3.3 ± 0.4	3.9 ± 0.7	4.7 ± 0.2	0.8 ± 0.1
**Glucose** (mmol/L)	S-A	1.1 ± 0.0	0.4 ± 0.1	0.9 ± 0.1	1.7 ± 0.2	1.2 ± 0.1
	L-A	0.9 ± 0.1	0.4 ± 0.1	0.5 ± 0.1	1.6 ± 0.1	1.0 ± 0.1

Post values are times after umbilical cord occlusion. S-A = Sal-Asp (saline infusion + saline boluses + 15 minutes of umbilical cord occlusion, n = 9); L-A = LPS-Sham (LPS infusion + LPS boluses + 15 minutes of umbilical cord occlusion, n = 8). LPS, lipopolysaccharide; paCO_2_, fetal arterial pressure of CO_2_ (mmHg); paO_2_, fetal arterial pressure of oxygen (mmHg). § *P* <0.05 versus Sal-Asp. Data are mean ± SEM.

### Oligodendrocytes

**Figure 2:**
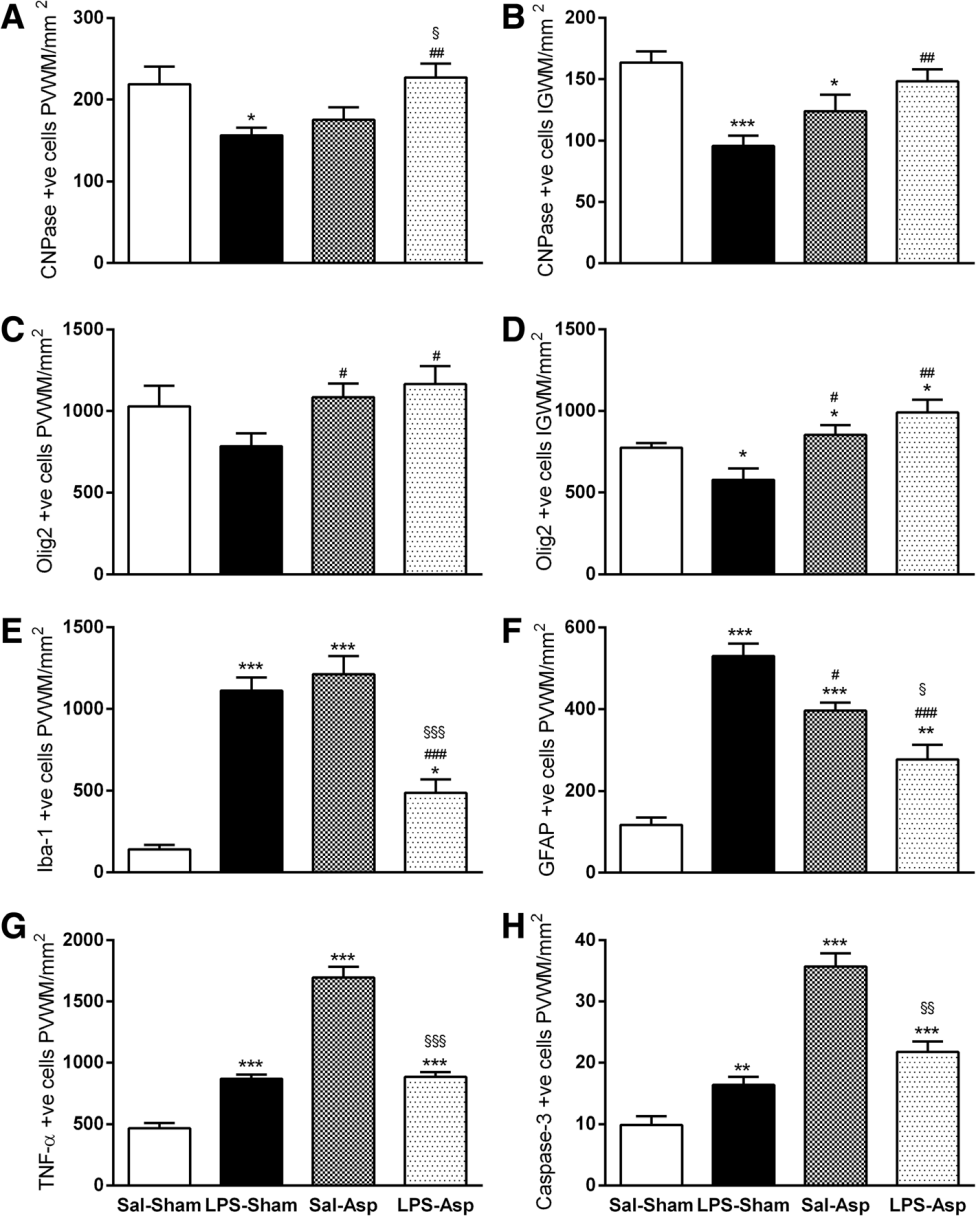
**Changes in numbers of immunohistochemically labeled white matter cells.** Bar graphs showing numbers of CNPase positive cells in the PVWM **(A)** and IGWM **(B)**, Olig2 positive cells in the PVWM **(C)** and IGWM **(D)**, Iba-1 positive cells **(E)** and GFAP positive cells in the PVWM **(F)**, TNF-α positive cells in the PVWM, **(G)** and caspase-3 positive cells in the PVWM **(H)**. *P <0.05, **P <0.01, ***P <0.001 versus Sal-Sham controls, # *P* <0.05 versus LPS-Sham, § *P* <0.05 versus Sal-Asp. Data are mean ± SEM. Asp, asphyxia; CNPase, 2′,-3′-Cyclic-nuceotide 3′-phosphodiesterase; GFAP, glial fibrillary acidic protein; Iba-1, ionized calcium-binding adapter molecule-1; IGWM, intragyral white matter; LPS, lipopolysaccharide; Olig2, Oligodendrocyte transcription factor-2; PVWM, periventricular white matter; TNF-α, tumor necrosis factor-α; Sal, saline.

**Figure 3:**
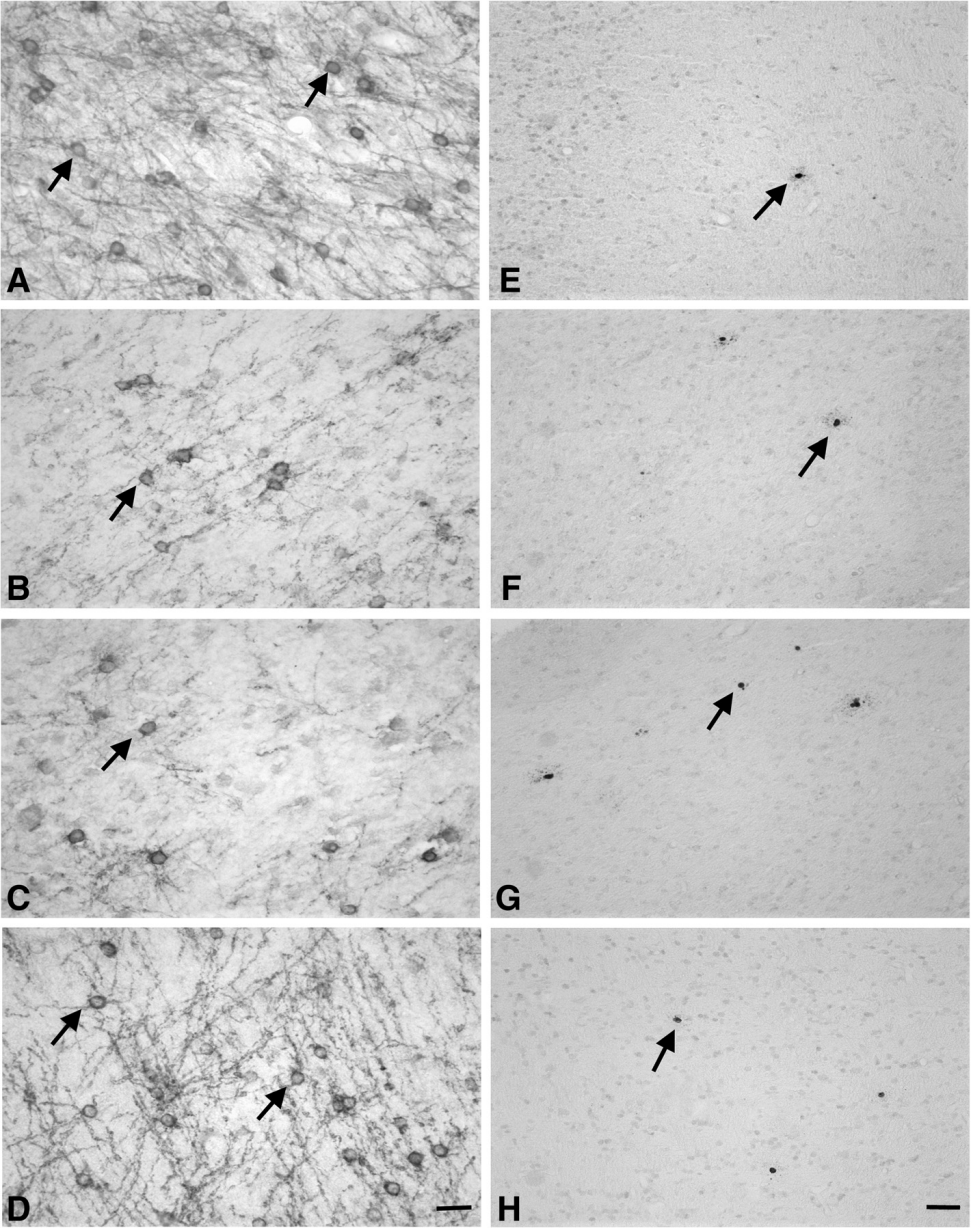
**Photomicrographs of immature and mature oligodendrocytes and caspase-3 labeled apoptotic cells in the PVWM.** Photomicrographs showing CNPase immunolabeling **(A-D)**, and cleaved caspase-3 (ASP175) immunolabeling **(E-H)** in the PVWM in Sal-Sham (row 1; **A**, **E**), LPS-Sham (row 2; **B**, **F**), Sal-Asp (row 3; **C**, **G**) and LPS-Asp (bottom; **D**, **H**). Arrows show examples of labeled cells. Note the reduction in CNPase positive cells and increase in activated caspase-3 labelling after LPS-Sham and Sal-Asp. Surviving CNPase positive cells show stunted processes. LPS-Asp treatment was associated with recovery of CNPase positive immature and mature oligodendrocytes to Sal-Sham values, and reduced numbers of activated caspase-3 positive cells compared with Sal-Asp but not LPS-Sham. Scale bar = 20 μm. Asp, asphyxia; CNPase, 2′,-3′-Cyclic-nuceotide 3′-phosphodiesterase; LPS, lipopolysaccharide; PVWM, periventricular white matter; Sal, saline.

LPS-Sham treatment was associated with a significant reduction in the number of CNPase positive oligodendrocytes in the PVWM and IGWM (*P* <0.05, Figures 2 and 3). Sal-Asp was associated with a significant overall loss of CNPase positive oligodendrocytes compared to Sal-Sham; *post hoc* tests suggested that there was a significant reduction in the IGWM (*P* <0.05), but not in the PVWM. Morphologically, many CNPase positive oligodendrocytes in the LPS-Sham and Sal-Asp groups showed a reduction in the number of processes and axonal contacts (Figure 3). In contrast, the LPS-Asp group was not significantly different from Sal-Sham, with significantly more CNPase positive oligodendrocytes than LPS-Sham in both regions overall (*P* <0.01).

LPS-Sham was associated with a significant reduction in the numbers of Olig2 positive oligodendrocytes in the IGWM (*P* <0.05 versus Sal-Sham, Figures 2 and 3) but not in the PVWM. Sal-Asp and LPS-Asp were associated with a significant increase in Olig2 positive oligodendrocytes compared to Sal-Sham (*P* <0.05). Further, both Sal-Asp (*P* <0.05) and LPS-Asp (*P* <0.01) were associated with increased numbers of Olig2 positive oligodendrocytes compared to LPS-Sham.

### Apoptotic cell death in PVWM

There was a significant increase in the number of activated caspase-3 positive apoptotic cells in the PVWM in the LPS-Sham group compared to Sal-Sham (*P* <0.01, Figures 2 and 3). Sal-Asp was associated with greater numbers of caspase-3 positive cells than all other groups (*P* <0.001). In contrast, the LPS-Asp group showed reduced numbers of caspase-3 positive cells compared to Sal-Asp, but was similar to the LPS-Sham group and still increased compared to Sal-Sham.

### Iba-1 positive microglia

**Figure 4:**
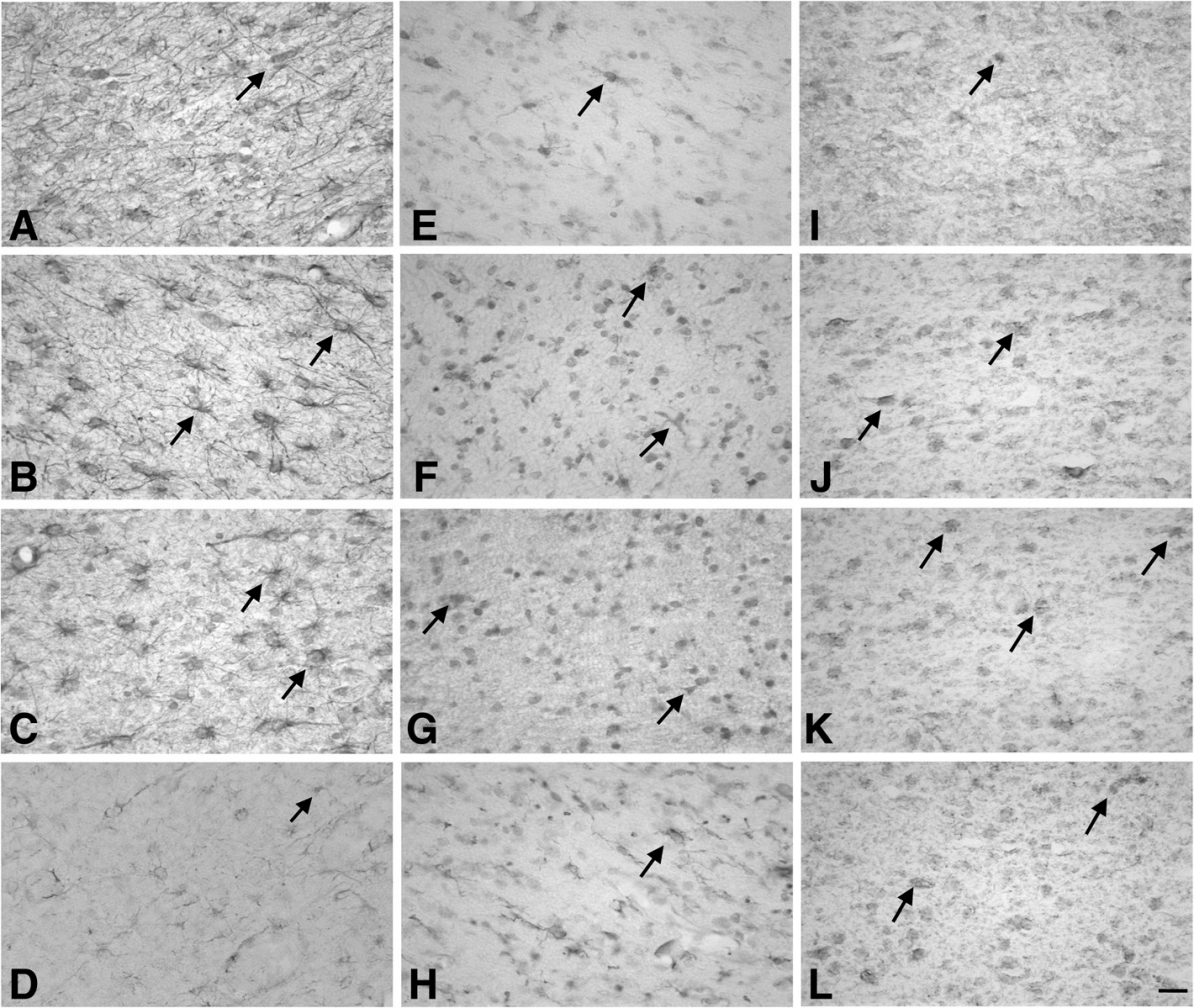
**Photomicrographs showing astrocytes, microglia and TNF-α labeled cells in the PVWM.** Photomicrographs showing glial fibrillary acidic protein (GFAP) (First column; **A-D**), Iba-1 (Second column; **E-H**), and tumor necrosis factor (TNF)-α immunolabeling (Third column; **I-L**) in the PVWM in Sal-Sham (row 1; **A**, **E**, **I**), LPS-Sham (row 2; **B**, **F**, **J**), Sal-Asp (row 3; **C**, **G**, **K**) and LPS-Asp (bottom; **D**, **H**, **L**). Arrows show examples of labeled cells. Note the robust induction of Iba-1 positive reactive microglia and GFAP positive reactive astrocytes after LPS-Sham treatment and Sal-Asp treatment, which was significantly attenuated in the LPS-Asp group. The LPS-Asp group showed astrocytes with thinner bodies and fewer processes than LPS-Sham and Sal-Asp. Scale bar = 20 μm. Asp, asphyxia; GFAP, glial fibrillary acidic protein; Iba-1, ionized calcium-binding adapter molecule-1; LPS, lipopolysaccharide; PVWM, periventricular white matter; Sal, saline; TNF-α: tumor necrosis factor-α.

Ramified microglia were predominantly present in the PVWM of Sal-Sham controls. LPS treatment and occlusion were independently associated with increased numbers of reactive microglia, larger cell bodies and thicker processes, and patchy infiltration of the white matter tracts (Figures 2 and 4). The area of the PVWM showing reactive microglial infiltration was significantly greater (*P* <0.001) in the LPS-Sham, Sal-Asp, and LPS-Asp groups compared to Sal-Sham (Sal-Sham 1.3 ± 1.2%; LPS-Sham 15.5 ± 1.0%; Sal-Asp 19.6 ± 5.0%, and LPS-Asp 12.9 ± 5.8%). Consistent with this, LPS-Sham and Sal-Asp were both associated with a significant increase in the numbers of microglia in the PVWM compared to Sal-Sham (*P* <0.001). In contrast, LPS-Asp was associated with reduced microglial induction compared to LPS-Sham and Sal-Asp (*P* < 0.001, Figures 2 and 4).

### Astroglial responses in the PVWM

LPS-Sham and Sal-Asp were associated with increased astrogliosis compared to Sal-Sham (*P* <0.01, Figures 2 and 4). In contrast, there was a significant reduction in reactive astrocytes in the LPS-Asp group compared to LPS-Sham and Sal-Asp (*P* <0.05).

### Induction of TNF-α

LPS-Sham and Sal-Asp were both associated with a significant increase in the number of cells expressing TNF-α in the PVWM (*P* <0.001, Figures 2 and 4) compared to Sal-Sham. The number of TNF-α positive cells was reduced in the LPS-Asp group compared to Sal-Asp (*P* <0.001).

### Cytokine and cortisol analysis

**Figure 5:**
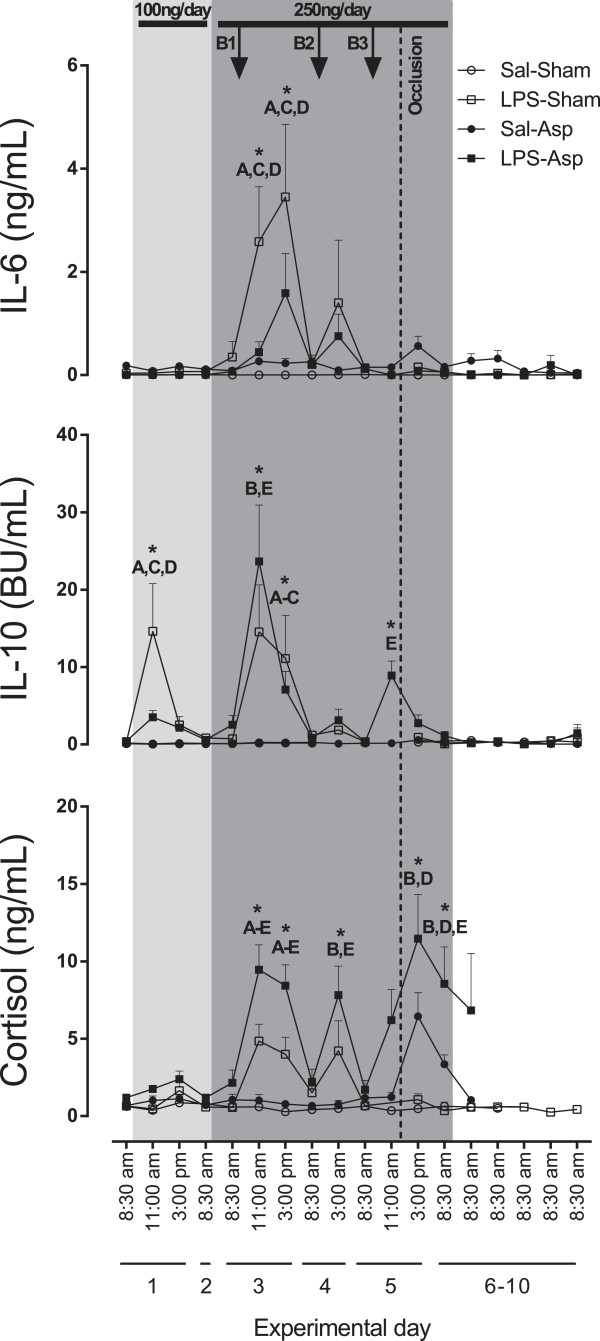
**Time sequence of changes in fetal plasma IL-6, IL-10 and cortisol concentrations.** The horizontal bars at the top of the figure show the chronic infusions of LPS or saline. The arrows represent the times of the three LPS bolus doses (B1, B2 and B3). The vertical dashed line shows the time of the 15 minute period of umbilical cord occlusion in the asphyxia groups. Data were not available in the LPS-Sham group for the 11 am time point on day five. Data are mean ± SEM. *P <0.05. A) Sal-Sham versus LPS-Sham; B) Sal-Sham versus LPS-Asp; C) LPS-Sham versus Sal-Asp; D) LPS-Sham versus LPS-Asp; E) Sal-Asp versus LPS-Asp. Asp, asphyxia; IL, interleukin; LPS, lipopolysaccharide; Sal, saline.

Low-dose, chronic LPS administration was associated with a transient increase in IL-10 in the LPS-Sham group compared to the Sal-Sham group (*P* <0.05, Figure 5). The first LPS bolus was associated with a significant increase in IL-6 in the LPS-Sham group compared to all other groups (*P* <0.05) and an apparent trend towards an increase in the LPS-Asp group compared to Sal-Asp (*P* = 0.09). A significant increase in IL-10 was seen in the LPS-Asp group compared to Sal-Sham and Sal-Asp, and in the LPS-Sham group compared to the Sal-Sham and Sal-Asp groups (*P* <0.05). The first bolus was also associated with a significant increase in cortisol levels in the LPS-Sham group and a significantly greater increase in the LPS-Asp groups compared to Sal-Sham (*P* <0.05). A similar increase in cortisol was seen after the second and third LPS boluses. A significant increase in cortisol was seen following asphyxia in the LPS-Asp group compared to all other groups. There were no further significant changes in IL-6 or IL-10 after the second and third bolus, other than a significant increase in IL-10 in the LPS-Asp group compared to Sal-Asp after the third bolus, prior to the onset of asphyxia (*P* <0.05). There were no differences between the Sal-Sham and Sal-Asp groups at any time point.

## Discussion

This study has demonstrated that acute-on-chronic LPS administration conditioned the preterm fetal sheep brain to reduce white matter injury from subsequent asphyxia. Intriguingly, this study also showed for the first time that asphyxia also conditioned the brain to reduce brain injury from preceding LPS exposure. Acute-on-chronic LPS administration was associated with significant loss of immature and mature oligodendrocytes and marked neuroinflammation and astrogliosis [17]. Complete umbilical cord occlusion for 15 minutes was associated with mild injury, including loss of immature and mature oligodendrocytes in the intragyral white matter and increased inflammation and astrogliosis. In contrast, animals exposed to both LPS and asphyxia showed marked attenuation of white matter inflammation and astrogliosis, with improved survival of immature and mature oligodendrocytes in the white matter compared to LPS exposure or asphyxia alone.

The present findings add to the evidence that there is substantial crossover in the mechanisms of infection and hypoxia-ischemia-related brain injury [25]. In contrast with studies of single acute doses of LPS that typically suggest that prior exposure to LPS increases the severity of later HI injury, the present study suggests a protective effect. The most likely reason for this difference is that continued exposure to LPS induced both self-tolerance and cross-tolerance, as found in both the whole animal [25] and even in isolated monocytes after chronic or repeated exposure to endotoxin *in vitro *[26,27]. This difference from previous neonatal studies is unlikely to reflect earlier exposure alone, since Eklind *et al. *[9] found marked sensitization when HI was induced 72 hours after exposure to LPS. Further, the present study used umbilical cord occlusion to induce asphyxia, with profound hypoxia, metabolic acidosis and hypotension, rather than carotid ligation with moderate hypoxia as used in neonatal rat studies. Nevertheless, adult rat studies have reported preconditioning with LPS before focal or global ischemia [25]. Finally, although we cannot rule out species differences, studies in adult rodents also suggest that LPS can protect against subsequent ischemic insults induced 48 hours or more after LPS [25].

Both acute-on-chronic LPS and asphyxia were associated with an overall loss of immature and mature (CNPase positive) oligodendrocytes in the white matter tracts, while LPS but not asphyxia was also associated with an overall reduction in total numbers of (Olig2 positive) oligodendrocytes. Further, both LPS and asphyxia were associated with a similar induction of microglia and astrogliosis. However, when combined as in the present study, LPS and asphyxia were associated with mutual protection, with significantly more immature and mature oligodendrocytes and reduced inflammation and astrogliosis, with a reduction in ongoing apoptosis compared with asphyxia alone.

This combination of greater total numbers of oligodendrocytes after asphyxia with the reduction in immature and mature oligodendrocytes in the present study denotes a relative increase in numbers of pre-oligodendrocytes in the white matter tracts. The oligodendroglia proliferative response to injury is almost entirely mediated by progenitor cells [28]. Thus, these data suggest reduced maturation into mature oligodendrocytes combined with significant proliferation of new pre-oligodendrocytes. This is consistent with data in the neonatal rat and the preterm fetal sheep that showed initial degeneration of pre-oligodendrocytes, that was offset by proliferation of pre-oligodendrocytes which, however, fail to mature after HI [29,30]. Similar evidence for the maturational arrest of pre-oligodendrocytes is seen in human neonatal white matter injury at postmortem [2].

In turn, the finding of extensive white matter astrogliosis in the present study is consistent with both clinical and experimental studies [2,29,30] and with increasing evidence that reactive astrocytes actively inhibit oligodendrocyte maturation [3]. In the present study the combination of asphyxia and inflammation was unexpectedly associated with restoration of the immature and mature oligodendrocytes to near-saline-sham values, with increased total oligodendrocyte number. The finding of reduced microglial induction and astrogliosis suggests that LPS pre-exposure reduced asphyxia-induced loss of pre-oligodendrocytes and that the combination attenuated the overall inflammatory reaction that was seen after both asphyxia and inflammation alone. Further longterm studies will be important to determine whether this arrest of maturation after five days recovery from asphyxia and the apparent improvement with LPS pre-exposure in the present study are persistent.

Although many mechanisms have been implicated in both tolerance and sensitization between infection and/or inflammation and HI, as seen in the present study, both asphyxia and inflammation are associated with a marked inflammatory response. The toll-like receptors that mediate innate immune responses can also sense cell damage after ischemia, and are present on neural microglia, oligodendrocytes, and neurons, as recently reviewed [25]. Self-induced tolerance to LPS is well recognized in many settings, and likely contributes to the greater cardiovascular tolerance and survival to high-dose LPS after chronic low-dose exposure as previously reported [17]. Alternatively, there is evidence that LPS can upregulate anti-inflammatory interferons in adult rodents [31].

The most intriguing aspect of this study is that protection against LPS-associated brain injury was seen when asphyxia was induced after the fetuses had received three high-dose boluses of LPS, although before the end of the chronic low-dose infusion. This striking finding denotes that much of the LPS-induced damage evolved over the six days after the last high-dose bolus. There is evidence for such postconditioning in other settings. For example, in adult mice, mild cerebral ischemia induced immediately, two minutes, or three hours after severe middle cerebral artery occlusion was shown to be neuroprotective and associated with reduced inflammation [32]. Postconditioning has also been shown to be neuroprotective in rodent models of global cerebral ischemia, both in terms of a reduction in behavioral deficits and neuronal death in the hippocampus and parietal cortex [33]. Previous experiments in rodent models suggest that the combination of preconditioning and postconditioning offers no synergistic protection, likely due to overlapping mechanisms of action [34,35]. However, in these studies, pre- and postconditioning were both induced by ischemia. In the current study, the synergistic neuroprotection may suggest different mechanisms of action for LPS-induced pre conditioning and ischemia postconditioning [36].

Alternatively, it is possible that counter-regulatory responses may have contributed in part to conditioning. Neuroprotection with LPS administration 24 hours before HI in seven-day-old rats was associated with upregulation of corticosterone and changes in gene expression, particularly those related to immune and inflammatory processes [37]. In the current study there was a significant increase in plasma cortisol levels after asphyxia in the LPS-Asp group, which may have contributed to pre conditioning. Further, we observed an increase in the anti-inflammatory cytokine IL-10 before asphyxia. IL-10 can reduce hypoxia-induced hyperexcitability in hippocampal slice neurons [38], and is an essential mediator of astroglial pre-conditioning to mild oxidative stress in culture [39]. Sevoflurane-induced postconditioning of global cerebral ischemia in adult rats is associated with suppression of pro-inflammatory cytokines and increased IL-10 [40]. Similarly, global cerebral ischemia in adult rats was associated with increased pro-inflammatory cytokines and decreased IL-10, which was reversed by mild ischemic preconditioning [41]. Thus, it is possible that the combination of greater cortisol and anti-inflammatory cytokines may have contributed in part to the decreased microglial and astrocyte activation seen with the combination of LPS and asphyxia compared to either LPS or asphyxia alone.

In conclusion, this study highlights a novel phenomenon of synergistic neuronal and white matter protection with LPS-preconditioning and ischemic postconditioning in preterm fetal sheep. Although exposure to infection and/or inflammation is associated with adverse outcomes, it may be relevant that prophylactic maternal antibiotics before preterm labor with intact membranes not only do not improve outcomes, but there is increasing evidence that they may be associated with increased risk of death and disability [42]. We speculate that in the context of the very high risks of neurodevelopmental impairment to which very preterm infants are exposed, that a little bit of inflammation may even be beneficial. Further studies of how the nature and timing of infection and/or inflammation modulate HI injury are needed to help understand their role in preterm brain injury.

## Abbreviations

Asp: asphyxia; CNPase: 2′,-3′-Cyclic-nuceotide 3′-phosphodiesterase; GFAP: Glial fibrillary acidic protein; HI: Hypoxia-ischemia; Iba-1: Ionized calcium-binding adapter molecule-1; IGWM: Intragyral white matter; IL: Interleukin; LPS: Lipopolysaccharide; NO: Nitric oxide; Olig2: Oligodendrocyte transcription factor-2; PBS: Phosphate-buffered saline; PVWM: Periventricular white matter; Sal: Saline; TNF-α: Tumor necrosis factor-α.

## Competing interests

The authors declare that there are no competing interests.

## Authors’ contributions

LB and AJG conceptualized the study. LB, AJG, LGH and JOD designed the protocol and setup the experimental preparations. LGH, JOD, CAL, LCB carried out experiments, and histology preparation. MF and CAL analyzed endocrine samples. LGH and SM analyzed immunohistochemistry. AJG, JOD and LB provided critical interpretation. LGH, AJG and SM drafted the paper. All authors contributed to editing the paper and have read and approved the final version of the manuscript.
